# Supplementary material for: Association of poverty-income ratio with cardiovascular disease and mortality in cancer survivors in the United States
Source: PLoS One. 2024 Jul 5;19(7):e0300154. doi: 10.1371/journal.pone.0300154 (PMC11226125; doi:10.1371/journal.pone.0300154)
Supplement: S1 File — (DOCX) [file pone.0300154.s001.docx]

**S1 Table.** **Descriptive Characteristics of the Cancer Survivors (Aged≥18 Years) by CVD-Status (NHANES 2003-2014)**

|  | CVD status | | | *P*-value ^a^ |
| --- | --- | --- | --- | --- |
|  | No-CVD | Pre-existing CVD | Post-acquired CVD |  |
| Unweighted sample size (n) | 1922 | 211 | 331 |  |
| Weighted population size (N) | 10,470,261 | 802,918 | 1,310,741 |  |
| Weighted population prevalence (%) ^b^ | 6.58 (6.20, 6.96) | 0.50 (0.42, 0.59) | 0.82 (0.69, 0.96) |  |
| Average follow-up years | 6.12 (5.97, 6.26) | 5.51 (5.09, 5.94) | 5.15 (4.82, 5.48) | <.001 |
| Unweighted deaths (n) | 317 | 78 | 132 |  |
| Age (years) | 60.07 (59.14, 61) | 70.99 (69.30, 72.67) | 70.44 (68.71, 72.17) | <.001 |
| Years since first diagnosed with cancer | 11.29 (10.56, 12.02) | 5.01 (4.23, 5.79) | 15.66 (14.19, 17.13) | <.001 |
| Sex (%) |  |  |  | <.001 |
| Male | 40.2 (37.79, 42.6) | 66.39 (58.25, 74.52) | 45.67 (39.54, 51.8) |  |
| Race/ethnicity (%) |  |  |  | <.001 |
| Non-Hispanic White | 88.13 (86.32, 89.94) | 87.47 (83.26, 91.69) | 90.19 (87.34, 93.05) |  |
| Non-Hispanic Black | 5.01 (3.95, 6.06) | 8.73 (5.32, 12.14) | 5.35 (3.42, 7.29) |  |
| Mexican | 2.09 (1.38, 2.80) | 1.19 (0.19, 2.19) | 1.93 (0.42, 3.43) |  |
| Others | 4.77 (3.62, 5.93) | 2.60 (0.63, 4.57) | 2.53 (1.16, 3.90) |  |
| Types of Cancer (%) ^c^ |  |  |  | <.001 |
| Obesity-Related IARC | 10.03 (8.29, 11.77) | 4.98 (1.49, 8.47) | 8.94 (5.68, 12.20) |  |
| Tobacco-Related IARC | 12.38 (10.35, 14.40) | 6.70 (2.58, 10.82) | 12.62 (7.15, 18.10) |  |
| Breast | 15.36 (13.53, 17.19) | 10.91 (6.39, 15.42) | 15.27 (9.82, 20.72) |  |
| Lung | 1.81 (1.13, 2.48) | 3.23 (0.00, 6.82) | 2.07 (0.00, 4.26) |  |
| Colon | 4.41 (3.32, 5.5) | 6.55 (3.47, 9.64) | 8.29 (4.93, 11.65) |  |
| Prostate | 7.43 (6.07, 8.79) | 21.87 (14.04, 29.69) | 9.40 (6.02, 12.79) |  |
| Melanoma | 7.11 (5.45, 8.77) | 7.93 (3.41, 12.44) | 5.67 (2.72, 8.62) |  |
| Leukemia & Lymphoma | 3.40 (2.35, 4.44) | 5.14 (0.82, 9.45) | 1.68 (0.00, 3.36) |  |
| All others | 38.08 (35.44, 40.73) | 32.7 (23.42, 41.98) | 36.05 (29.13, 42.97) |  |
| Education (%) |  |  |  | <.001 |
| <High school | 12.19 (9.92, 14.46) | 21.92 (14.87, 28.97) | 27.46 (21.49, 33.44) |  |
| High school diploma or equivalent | 20.71 (18.15, 23.28) | 26.22 (19.96, 32.48) | 23.92 (17.81, 30.03) |  |
| ≥College or above | 67.09 (63.73, 70.46) | 51.86 (44.81, 58.91) | 48.62 (40.67, 56.57) |  |
| Marital Status (%) |  |  |  | .004 |
| Married or living with partner | 68.73 (65.91, 71.55) | 63.68 (54.52, 72.84) | 57.18 (49.98, 64.38) |  |
| Smoking status (%) |  |  |  | .613 |
| Currently smoking | 17.07 (14.72, 19.41) | 14.32 (8.26, 20.37) | 18.97 (12.8, 25.15) |  |
| Health insurance (%) |  |  |  | .639 |
| Not-covered | 20.08 (17.05, 23.11) | 20.83 (13.66, 28.00) | 17.53 (11.95, 23.10) |  |
| Alcohol consumption (%) |  |  |  | .004 |
| ≥12 drinks/year | 74.78 (71.91, 77.64) | 72.26 (65.65, 78.88) | 64.76 (58.08, 71.44) |  |
| Body mass index (%) |  |  |  | .393 |
| <25 kg/m^2^ | 30.87 (28.41, 33.33) | 27.05 (20.57, 33.53) | 24.66 (18.98, 30.34) |  |
| 25 - <30 kg/m^2^ | 34.73 (31.53, 37.92) | 36.64 (28.03, 45.25) | 37.39 (32.01, 42.78) |  |
| ≥30 kg/m^2^ | 34.40 (31.96, 36.85) | 36.31 (27.68, 44.95) | 37.95 (31.05, 44.85) |  |
| Walking difficulty score | 1.15 (1.13, 1.18) | 1.30 (1.25, 1.36) | 1.39 (1.32, 1.46) | <.001 |
| HEI 2015 | 53.13 (52.19, 54.07) | 52.49 (50.83, 54.15) | 53.36 (51.37, 55.34) | .725 |
| MVPA levels (%) |  |  |  | <.001 |
| I-MVPA (<150 mins/wk) | 52.79 (50.28, 55.3) | 44.82 (36.91, 52.73) | 36.19 (29.51, 42.87) |  |

Abbreviation: CVD = cardiovascular disease; I-MVPA = insufficient moderate and vigorous-intensity physical activity (<150 minutes of MVPA per week); HEI = healthy eating index

Values are the mean (95% CI) and the percentage (95% CI) for continuous and categorical variables, respectively, estimated after accounting for the complex sampling design of the NHANES.

^a^ P-value is for the between-group differences estimated from a linear regression model for a continuous variable and Rao-Scott x2 test of independence for a categorical variable.

^b^ Weighted population % indicates the prevalence estimates among US adults aged ≥18 years.

^c^ 1^st^ diagnosed cancer type, if multiple cancers were reported.

**S2 Table.** **The Multivariate Cox Regression Model Examining Independent Associations of Poverty-Income Ratio and CVD Status with All-Cause Mortality Among Cancer Survivors^a^**

|  | Hazard ratio (95% CI) | *P*-value |
| --- | --- | --- |
| Gender |  |  |
| Male | 1.90 (1.44, 2.52) | <.001 |
| Female | Reference |  |
| Age (years) | 1.09 (1.07, 1.10) | <.001 |
| Race/ethnicity |  |  |
| Non-Hispanic White | Reference |  |
| Non-Hispanic Black | 1.45 (1.02, 2.07) | .038 |
| Mexican | 0.79 (0.44, 1.40) | .408 |
| Others | 0.66 (0.34, 1.27) | .204 |
| Cancer types |  |  |
| Obesity-Related IARC | 0.87 (0.53, 1.44) | .589 |
| Tobacco-Related IARC | 0.97 (0.65, 1.45) | .902 |
| Breast | 1.01 (0.71, 1.45) | .839 |
| Lung | 2.51 (1.11, 5.70) | .046 |
| Colon | 1.02 (0.70, 1.49) | .874 |
| Prostate | 0.87 (0.63, 1.19) | .297 |
| Melanoma | 0.78 (0.5, 1.22) | .229 |
| Leukemia, Lymphoma | 1.90 (1.06, 3.41) | .027 |
| All others | Reference |  |
| Marital status |  |  |
| Married or living with partner | 0.74 (0.58, 0.94) | .015 |
| Others | Reference |  |
| Smoking status |  |  |
| Not smoking | Reference |  |
| Currently smoking | 1.98 (1.39, 2.81) | <.001 |
| Body mass index |  |  |
| <25 kg/m^2^ | Reference |  |
| 25 - <30 kg/m^2^ | 0.79 (0.64, 0.97) | .026 |
| ≥30 kg/m^2^ | 0.77 (0.57, 1.04) | .089 |
| MVPA levels |  |  |
| I-MVPA (<150 mins/wk) | 1.34 (1.05, 1.71) | .019 |
| S-MVPA (≥150 mins/wk) | Reference |  |
| Walking difficulty score | 1.86 (1.54, 2.32) | <.001 |
| PIR |  |  |
| Highest tertile | Reference |  |
| Middle tertile | 1.49 (1.04, 2.14) | .001 |
| Lowest tertile | 1.71 (1.24, 2.36) | .031 |
| CVD-Status |  |  |
| No-CVD | Reference | .003 |
| Pre-existing CVD | 1.35 (1.00, 1.84) | .050 |
| Post-acquired CVD | 1.67 (1.30, 2.15) | <.001 |

Abbreviation: CVD = cardiovascular disease; PIR = poverty-income ratio

^a^ This supplementary table complements the results presented in Table 3 in the main body.

^b^ Hazard ratios are estimated from a multivariate Cox proportional hazard regression model, which includes a single combined variable (PIR-by-CVD status), while adjusting for age, sex, race/ethnicity, cancer type, marital status, smoking, physical activity, body mass index, and walking difficulty score that were retained by the backward elimination method (*P*<.20).

**S3 Table.** **Combined Associations of Poverty-Income Ratio and CVD Status with All-Cause Mortality Among Cancer Survivors^a^**

| PIR | CVD Status | Hazard ratio (95% CI) ^b^ | | | | | | | |
| --- | --- | --- | --- | --- | --- | --- | --- | --- | --- |
| Highest tertile | No-CVD | **Ref** | - | - | - | - | - | - | - |
|  | Pre-existing CVD | 0.79  (0.38, 1.68) | **Ref** | - | - | - | - | - | - |
|  | Post-acquired CVD | 1.54  (0.91, 2.62) | 1.94  (0.79, 4.75) | **Ref** | - | - | - | - | - |
| Middle tertile | No-CVD | 1.27  (0.88, 1.82) | 1.60  (0.73, 3.48) | 0.82  (0.44, 1.53) | **Ref** | - | - | - | - |
|  | Pre-existing CVD | 2.10  (1.24, 3.54) | 2.64  (1.06, 6.61) | 1.36  (0.63, 2.94) | 1.66  (1.05, 2.61) | **Ref** | - | - | - |
|  | Post-acquired CVD | 2.71  (1.69, 4.35) | 3.42  (1.46, 8.01) | 1.76  (0.91, 3.40) | 2.14  (1.49, 3.07) | 1.29  (0.78, 2.13) | **Ref** | - | - |
| Lowest tertile | No-CVD | 1.72  (1.21, 2.45) | 2.17  (0.98, 4.79) | 1.12  (0.69, 1.83) | 1.36  (0.91, 2.03) | 0.82  (0.48, 1.41) | 0.64  (0.43, 0.93) | **Ref** | - |
|  | Pre-existing CVD | 2.38  (1.37, 4.14) | 3.00  (1.29, 7.01) | 1.55  (0.74, 3.23) | 1.88  (1.18, 3.00) | 1.14  (0.62, 2.10) | 0.88  (0.57, 1.35) | 1.38  (0.85, 2.25) | **Ref** |
|  | Post-acquired CVD | 2.17  (1.27, 3.71) | 2.74  (1.17, 6.38) | 1.41  (0.76, 2.63) | 1.71  (1.03, 2.84) | 1.04  (0.55, 1.96) | 0.80  (0.50, 1.29) | 1.26  (0.84, 1.90) | 0.91  (0.49, 1.69) |

Abbreviation: CVD = cardiovascular disease; PIR = poverty-income ratio

^a^ This supplementary table complements the results presented in Table 4 in the main body by providing hazard ratios estimated using different reference categories.

^b^ Hazard ratios are estimated from a Cox proportional hazard regression model, which includes a single combined variable (PIR-by-CVD status), while adjusting for age, sex, race/ethnicity, cancer type, marital status, smoking, physical activity, body mass index, and walking difficulty score that were retained by the backward elimination method (*P*<.20). The full model including the parameters estimated for study covariate is presented in Supplement Table 4.

**S4 Table.** **The Multivariate Cox Regression Model Examining Combined Associations of Poverty-Income Ratio and CVD Status with All-Cause Mortality Among Cancer Survivors^a^**

|  | Hazard ratio (95% CI) | *P*-value |
| --- | --- | --- |
| Gender |  |  |
| Male | 1.88 (1.42, 2.49) | <.001 |
| Female | Reference |  |
| Age (years) | 1.09 (1.07, 1.10) | <.001 |
| Race/ethnicity |  |  |
| Non-Hispanic White | Reference |  |
| Non-Hispanic Black | 1.41 (1.00, 2.01) | .053 |
| Mexican | 0.77 (0.43, 1.37) | .363 |
| Others | 0.68 (0.36, 1.29) | .233 |
| Cancer types |  |  |
| Obesity-Related IARC | 0.87 (0.53, 1.41) | .558 |
| Tobacco-Related IARC | 0.97 (0.65, 1.45) | .874 |
| Breast | 1.01 (0.71, 1.45) | .950 |
| Lung | 2.51 (1.11, 5.70) | .027 |
| Colon | 1.02 (0.70, 1.49) | .916 |
| Prostate | 0.87 (0.63, 1.19) | .368 |
| Melanoma | 0.78 (0.5, 1.22) | .281 |
| Leukemia, Lymphoma | 1.90 (1.06, 3.41) | .032 |
| All others | Reference |  |
| Marital status |  |  |
| Married or living with partner | 0.74 (0.58, 0.95) | .018 |
| Others | Reference |  |
| Smoking status |  |  |
| Not smoking | Reference |  |
| Currently smoking | 1.98 (1.39, 2.83) | <.001 |
| Body mass index |  |  |
| <25 kg/m^2^ | Reference |  |
| 25 - <30 kg/m^2^ | 0.79 (0.64, 0.97) | .026 |
| ≥30 kg/m^2^ | 0.77 (0.57, 1.04) | .086 |
| MVPA levels |  |  |
| I-MVPA (<150 mins/wk) | 1.33 (1.05, 1.70) | .021 |
| S-MVPA (>=150 mins/wk) | Reference |  |
| Walking difficulty score | 1.89 (1.54, 2.32) | <.001 |
| PIR & CVD status combined |  |  |
| Highest PIR tertile-No-CVD | Reference |  |
| Highest PIR tertile-Pre-existing CVD | 0.79 (0.38, 1.68) | .541 |
| Highest PIR tertile-Post-acquired CVD | 1.54 (0.91, 2.62) | .109 |
| Middle PIR tertile-No-CVD | 1.27 (0.88, 1.82) | .198 |
| Middle PIR tertile-Pre-existing CVD | 2.10 (1.24, 3.54) | .006 |
| Middle PIR tertile-Post-acquired CVD | 2.71 (1.69, 4.35) | <.001 |
| Lowest PIR tertile-No-CVD | 1.72 (1.21, 2.45) | .003 |
| Lowest PIR tertile-Pre-existing CVD | 2.38 (1.37, 4.14) | .002 |
| Lowest PIR tertile-Post-acquired CVD | 2.17 (1.27, 3.71) | .005 |

Abbreviation: CVD = cardiovascular disease; PIR = poverty-income ratio

^a^ This supplementary table complements the results presented in Table 4 in the main body and Supplement Table 2.

^b^ Hazard ratios are estimated from a multivariate Cox proportional hazard regression model, which includes a single combined variable (PIR-by-CVD status), while adjusting for age, sex, race/ethnicity, cancer type, marital status, smoking, physical activity, body mass index, and walking difficulty score that were retained by the backward elimination method (*P*<.20).
