# Supplementary material for: Association of poverty-income ratio with cardiovascular disease and mortality in cancer survivors in the United States
Source: PLoS One. 2024 Jul 5;19(7):e0300154. doi: 10.1371/journal.pone.0300154 (PMC11226125; doi:10.1371/journal.pone.0300154)
Supplement: Attachment — Submitted filename: 1_2. Response letter.docx [file pone.0300154.s002.docx]

Dear Reviewers,

Thank you so much for taking the time to read our paper and to give us feedback on this important topic. We have responded to the individual comments line-by-line below and hope the manuscript is now acceptable for publication.

**Reviewer #1**

Yogeswaran et al. have analyzed the interesting topic on the association of income, cancer, cardiovascular disease (CVD), and mortality. However, in this manuscript the relationship between introduction, the methods used, and interpretation of the results is not straightforward in my view. Here are the details, along with some other comments:

Thank you so much for your thoughtful comments. The present work was driven by the author(s)' genuine interest in health disparities in cardio-oncology, which have been significantly understudied in the literature. We have revised the manuscript to address the reviewer’s comments whenever possible by highlighting the gaps in income disparities in cardio-oncology, CVD prevalences, and the risk of all-cause mortality. Below, we have provided the point-by-point responses to the reviewer’s comments:

1. The analysis focuses on cancer survivors, as this group is known to have elevated CVD risks. These risks have been attributed to usual CVD risk factors and (adverse) effects of cancer therapy, so far, and become “a critical cause for morbidity and mortality”. (Which further morbidity, is it relevant here?) Although this points to CVD-related mortality, the endpoint defined in this analysis is all-cause mortality. Was no information available on causes of mortality? All-cause mortality introduces more complexity, and income is a risk factor for many causes of death. Thus, one would expect differences in all-cause mortality by income even when controlling for CVD. The authors do not sufficiently introduce or discuss these pathways (which covariates are supposed to be confounders, which one’s mediators?), and do not discuss different causes of mortality at all. Accordingly, the regression models are based on statistical significance rather than on knowledge about the pathways.

We have added a sentence (page 3, line 17) “ (15). Additionally, several forms of cancer therapy have been linked to long-term cardiovascular effects including coronary artery disease, heart failure, and vascular disease (16, 17).” The individual subtypes are not the focus of this article, as these have been highlighted in other large studies. Rather, we wish to emphasize the gaps in our knowledge of cardio-oncology, and the social determinants of health.

Regarding the mortality comment, we agree. The NHANES publicly available mortality data included nine primary causes of the death information. However, due to the relatively small sample size of the target population in the present study, there were insufficient numbers of cause-specific mortality cases. Therefore, we were unable to ascertain individual causes of mortality for this study. We have added this to the limitations section and methods section.

“Unfortunately, due to the limited sample size, we were unable to broaden our scope to include cause-specific mortality, including CVD-specific mortality. However, several large studies have reported that CVD is the most common cause of non-cancer death, likely secondary to cardiotoxicity(53, 54), allowing us to highlight that income is an important risk measure for adverse health outcomes in this population.”

We tested independent and combined associations of two primary independent variables, income levels and CVD status, with all-cause mortality while adjusting for the potential influences of study covariates including “age, sex, race/ethnicity, cancer type, marital status, smoking, physical activity, body mass index, and walking difficulty score.”, which have been consistently highlighted as common risk factors of mortality in the literature. We agree with the reviewer that exploring potential roles of such study covariates as confounders and mediators would be interesting and necessary steps to further elucidate the complexity of shared common risk factors between income, CVD, and all-cause mortality. However, determining such complex associations from the NHANES dataset, where all variables except mortality data were collected in a cross-sectional manner, could be challenging. Further, our goal was to show the association of income, CVD status and mortality in this cohort on a broad level; hopefully, future studies can determine the mechanistic pathways driving this association.

2. The discussion lacks comparison of the results with other studies, and especially with the NHANES-population without cancer. Are the differences by income in the ‘general’ NHANES population the same for ‘No-CVD’ and participants with CVD diagnosed before cancer?

We have added several sentences to page 9, starting at line 28.

“Prior studies have shown that low income is associated with poorer health behaviors, health status, and increase the risk of CVD in the general population [10, 11, 28-31]. Recently, Minhas et al observed significant associations between household income-tertile and adverse cardiovascular outcomes in NHANES participants [32]. In adjusted analysis, the prevalence of diabetes, hypertension, coronary artery disease, heart failure and stroke decreased in a stepwise manner from the highest to lower PIR[32]. In a separate NHANES analysis, lower PIR was associated with all-cause mortality independent of demographic, lifestyle, and clinical risk factors in the general population[33]. In our study of cancer survivors…”

It would be interesting to compare the income-CVD relationship among ‘general’ population with cancer survivors with different CVD status; however, we did not include a comparator group for those without cancer as our specific focus was to look at the association in those with a history of cancer, since this group is understudied compared to the general population.

3. In part, I could not completely understand how regression analysis was conducted, or to which results it refers: In the methods section, 2 models are introduced, the first includes (among others) both, income and CVD. But table 3 does not list the respective other variable? In my opinion, the mutual adjustment would be essential here. The second model should additionally include an interaction term (‘joint effect’), or does it refer to a further stratified variable as shown in table 4?

Valid point. We agree that our description of the statistical model was not clear. We have clarified this during this revision. Table 3 presents the results from the model including both PIR and CVD status as primary predictors of mortality while adjusting for study covariates. We have specified this in the method section “We first fitted a model including both PIR and CVD status to test independent associations on mortality risk.”. We have also clarified this under the note in Table 3 “Hazard ratios are estimated from a Cox proportional hazard regression model, which includes both PIR and CVD status, while adjusting for age, sex, race/ethnicity, cancer type, marital status, smoking, alcohol consumption, physical activity, body mass index, and walking difficulty score that were retained by the backward elimination method (*P*<.20).”.

Table 4 presents the results from a Cox regression model where a single combined predictor (income-by-CVD status, where ‘highest PIR tertile/No-CVD was a primary ‘reference’ group) was used as a primary predictor of mortality while adjusting for study covariates. Thus, the interaction term was deemed unnecessary and was not included in the model. We presented this in the method section as “The second model included the combined variable of PIR and CVD to test a joint association on mortality risk.”. We have also clarified this under the note in Table 4 “Hazard ratios are estimated from a Cox proportional hazard regression model, which includes a single combined variable (PIR-by-CVD status), while adjusting for age, sex, race/ethnicity, cancer type, marital status, smoking, alcohol consumption, physical activity, body mass index, and walking difficulty score that were retained by the backward elimination method (P<.20).”

We also provided additional supplement tables presenting the parameter estimates of the full models including study covariates retained. 4. Maybe related to this: I cannot follow the conclusion that income is a stronger predictor than CVD. Which results do support this?

Valid point. Our interpretation stating income as a stronger predictor of all-cause mortality than CVD status was based on the results from the combined Cox regression analysis that are presented in Table 4. The results showed that, when compared to the reference of no CVD in the highest tertile, no CVD in the lowest tertile has a 72% increased risk of mortality. In the same table, when compared to the highest tertile reference of no CVD, post-acquired CVD does not have a statistically significant difference in CVD risk. We have provided additional results about HRs relative to alternative reference group in the Supplement Table 3.

To address the reviewer’s comment, we have adjusted the wording in the results “In the follow-up multivariate Cox regression analysis testing the combined association of PIR and CVD status (Table 4), Similarly, additional comparisons with alternative reference groups (Supplement Table 3) demonstrated that, among cancer survivors with no CVD, we observed a 72% increase in risk in mortality between the lowest and highest PIR tertile (HR = 1.72; 95% CI = 1.69-4.35). Additionally, in cancer survivors with pre-existing CVD, the lowest and middle-PIR tertile groups continued to have a higher risk of mortality than the highest PIR tertile by 200% (HR = 3.00; 95% CI = 1.29-7.01) and 164% (HR = 2.64; 95% CI = 1.06, 6.61), respectively. These findings collectively suggest that PIR may be a potent predictor of mortality as pre-existing CVD and even a stronger predictor than post-acquired CVD.“

5. The terminology/language in the manuscript are partially not consistent and precise enough. E.g., income is measured on individual, household or area level, and there are absolute or relative measures, all of which may be differently associated with health outcomes. The manuscript does not seem to differentiate in this respect. Another example is use of terms like ‘independently associated’ all over the manuscript: it is often not clear, what exactly this means.

Thank you for pointing this out. I have adjusted the wording through the manuscript and have added a section in the limitations section to account for the PIR). PIR evaluates family income over the poverty thresholds, specific to family size by state and survey years.

The phrase 'independently associated' refers to the factor associated with the outcome while adjusting for other variables in the model. In the context of our study, as shown in Table 2, PIR and CVD status were both independently associated with all-cause mortality, mutually adjusted, in addition to other study covariates. We refer to this as being 'independently associated,' and we have clarified this in the method section “We first fitted a model including both PIR and CVD status to test independent associations on mortality risk.”

6. In the classification of cancer types, there are some overlaps. How did the authors decide to classify e.g. lung cancer? What was the procedure in case of more than one cancer diagnosis (also with respect to follow-up time?). This should be mentioned in the methods section.

We have moved this section from the covariates to the study population and added more description to answer this question:

“We considered cancer survivors to be those individuals who had been previously diagnosed with cancer and who had provided valid responses to questions regarding the types of cancer and ages when they were first diagnosed with those cancers. Cancer-type was grouped into the following categories: Obesity-related cancer, Tobacco-related cancer, and individual sub-types. Obesity-related was defined by the International Agency for Research on Cancer (IARC) to include esophageal, colorectal, gallbladder, pancreas, breast, uterine, ovarian, kidney, or thyroid cancer (21) and Tobacco-related IARC includes nasal cavity, paranasal sinus, nasopharynx, hypopharynx, oropharynx, larynx, esophageal, bladder, cervix, lung, stomach, liver, and myeloid leukemia(22), Individual cancer sub-types were separated into 1) Breast, 2) Lung, 3) Colon, 4) Prostate, 5) Melanoma, 6) Blood (Leukemia and Lymphoma), and 7) All other types of cancer. If multiple cancers are reported, the first diagnosed cancer type was considered as the primary cancer and used for categorization.”

We have also clarified this under the note in the Table 1 “^e^ 1^st^ diagnosed cancer type, if multiple cancers were reported.”

7. Overall, income is differently associated with different cancer types. The analysis does not consider this (probably due to small numbers), but it should at least be discussed.

Thank you for pointing this out. I have added a small section to the discussion (Page 11, starting at line 6)

“Additionally, while we observed that lower PIR is associated with a higher prevalence CVD and increased risk of all-cause mortality, underlying mechanisms are complex, and we are unable to ascertain the exact pathways leading to this association. All-cause mortality in cancer survivors encompasses both cancer-related mortality and non-cancer related mortality. Income may differentially impact different cancer subtypes and mortality risks, with most studies reporting that low household income is associated with worse outcomes [53, 54].”

8. Sex/gender is associated in every (biological, social/behavioural) respect. Therefore, I would suggest a stratified analysis by sex (at least supplementary).

Thank you for pointing this out. We agree this is important and may be of interest in future studies. However, our primary interest in this paper and analysis is to evaluate the general relationship between household income, cardiovascular risk, and mortality.

9. Is there a reference for the cut point (p=0.2) for backward elimination? It seems rather high, and possibly a reason why nearly all initial covariates are included.

Based on our understanding, there is no absolute threshold of P-value recommended for retaining study covariates when using the backward elimination method. The choice depends on various factors, including the total sample size, the number of study covariates, their potential role as influencing factors for either the independent variable (IV), dependent variable (DV), or both, and the parsimony of the model. Given the observational in nature of the NHANES, our goal was to develop a parsimonious prediction model while retaining potential covariates associated with all-cause mortality at a marginally significant level of 0.20 (Note: 'Marginally significant' is a subjective interpretation of the P-value applied only for model building in the present study). Although not perfect, this also could possibly alleviate potential selection bias for between-group comparisons (i.e., by PIR and/or CVD status). In the literature, there is still considerable inconsistency in selecting the threshold of P-value for backward elimination, ranging from 0.01 or lower to as high as 0.30 or higher.

In line with the reviewer’s suggestions, we have found several studies that subjectively define a P-value of 0.20 as a threshold for the backward elimination method:

<https://pubmed.ncbi.nlm.nih.gov/25430504/>

<https://pubmed.ncbi.nlm.nih.gov/27151893/>

<https://pubmed.ncbi.nlm.nih.gov/25415265/>

<https://pubmed.ncbi.nlm.nih.gov/15567629/>

And, although not ‘directly’ related to backward elimination for Cox regression model, in the study of Wang et al (2006) entitled “Determination of the Selection Statistics and Best Significance Level in Backward Stepwise Logistic Regression Qinggang Wang, John J. Koval,Catherine A. Mills & Kang-In David Lee”, the authors(s) recommend to use P-value between .20 and .40 as a stopping criterion for variable selection (https://www.tandfonline.com/doi/full/10.1080/03610910701723625).

Furthermore, we have generated a new supplementary table (Supplement Table 2 and 4) containing the estimated parameters for all study variables derived from the model presented in Table 3 and 4.

10. For mortality, follow-up years are only mentioned in methods, what was the starting point?

We have included the requested information in Table 1. The starting point of the follow-up is specified in the method section as the date of the interview.

11. Tables/figures:
- Table 1 should additionally include data on mortality, NHANES-waves, and follow-up years/person-years at risk.

We have incorporated the mortality data in Table 1 in addition to the follow-up years. The descriptive characteristics were not stratified by the NHAENS cycle as our goal was to combine these multiple cycle data into one cross-sectional data.

- In tables 3 and 4, there are columns with alternative reference groups and hyphens for some categories. Are results just omitted for some purpose?

The omitted categories represent the inverse of the relative risk between the same groups that have already been compared and presented. Thus, to avoid unnecessary confusion, we purposefully omitted these duplicate categories and presented this alternative comparisons in Supplement Table 3.

- Figure 2 could be omitted, if it shows the same results as table 3.

We removed Figure 2.

12. The presentation of results of table 2 in the text seems to be wrong and misleading: There is no general elevated prevalence in the lowest income tertile. I don’t see any common pattern overall.

The wording has been adjusted. We agree that it was confusing. “In Table 2, the prevalence of pre-existing and post-acquired CVD are reported. The cancer survivors in the lowest PIR tertile had the highest prevalence of pre-existing stroke (44.5% vs. 20-26% in the middle and highest tertile). The prevalence of post-acquired CVD, the number of years since first diagnosed with CVD, number having two or more cardiovascular conditions, heart failure, and stroke were higher in the lowest-tertile compared to the highest-tertile. However, these comparisons did not reach statistical significance. “

13. Numbers for middle income tertile are different in the text as compared to corresponding table (p 9 lines 19 and 21)

We only reported the lowest and highest tertiles in the text.

14. On page 12, authors note the ‘nationally representative’ US sample, and then state a limitation is that the ‘demographics in the actual cohort are representative of the US population’. This seems to be contradictory.

Sorry I have caught my error and have fixed this. I meant applicability outside of the US population.

**Reviewer #2**

In the work presented, the aim was to determine the relationship between CVD and income inequality among cancer survivors. In my view, the motivation for this project is not clearly stated in the introduction. Why is this correlation particularly interesting among cancer survivors? Wouldn't it be more interesting to compare this influence in people with and without cancer to understand whether it differs between the groups? Without taking into account people without cancer, I also think the statement that the increased cardiometabolic risk and vulnerability may be exacerbated in cancer survivors is not necessarily valid.

Thank you for your comments. I have changed the introduction section to highlight the importance of our question. We agree that this is important, but there are several studies focusing on the impact of income in patients without cancer in large population-based studies. In the future, it would be interesting to directly compare. At this point, there are large gaps in knowledge about how the social determinants of health impact cardiovascular risk and outcomes in cancer survivors as it has been an understudied population for this specific purpose. Our primary objective is to study how income impacts cancer survivors in NHANES.

Furthermore, the presentation in the tables with the results of the statistical models is not clear. I cannot understand what exactly was calculated, which covariates are included in the models and how the calculated models differ. As the full model results are not shown, it is not possible to assess the effects of the extensive adjustment variables.

We made substantial revisions in the methods and results sections, as well as the tables, to enhance the clarity of the statistical models and the interpretation of the results. Additionally, we created supplementary tables presenting parameter estimates for all variables included in the multivariate Cox regression models.

Moreover, I cannot always understand the conclusions drawn from the results (e.g., that low income is a stronger predictor of mortality than CVS). Looking at Figure 2, the two risk factors appear to me to be similarly strong.

As per the reviewer’s comments, we have updated the wording in the results and discussion to make it clearer.

Below are more detailed comments:
Abstract: The abstract should include more details on the methods used. At this point, the previously unexplained abbreviation "HR" could also be introduced.

The abstract has been updated to include more details.

Methods:
The inclusion and exclusion criteria could be presented in more detail (page 5). For example, was there an inclusion criterion regarding a minimum or maximum length of time since cancer diagnosis? Perhaps people with childhood cancer diagnoses have a different CVD risk than other cancer survivors? This should be discussed. Please also specify “valid responses to questions regarding the types of cancer and ages” (page 5, line 3).

The study population has been updated. We agree that these risks can be different. To allow adequate study size, we included all patients regardless of time since cancer diagnosis. In the future this would be an interesting sub-study using a different study population.

“We considered cancer survivors to be those individuals who had been previously diagnosed with cancer and answered “yes” to the question, “Has a doctor ever told you that you had cancer or malignancy?” Cancer sub-types were assessed through response to the question “What kind of cancer?”, and respondents were able to enter up to 3 kinds. Age when cancer was first diagnosed was assessed through a separate survey question(21, 22).”

Page 6: Please provide at least rudimentary information on the healthy eating index (e.g. range, are higher or worse values good, etc.).

A sentence has been added to account for this.

Page 7:
I do not understand the following sentence: „We particularly estimated the prevalence of cancer survivors with 2 or more CVDs and type of CVD (i.e., CAD, heart failure, and stroke) across the time when CVD was diagnosed (i.e., pre-existing before cancer diagnosis or post-acquired CVD after cancer diagnosis).”
How are multiple CVDs measured? I had understood the previous paragraph to mean that people are always assigned to one of three CVD groups. Please clarify.

Thank you for pointing this out. We have updated the wording to make this clearer.

“We reported the prevalence of cancer survivors with 2 or more CVDs (CAD, heart failure, and stroke) and individual CVD diagnosis (across the time when CVD was diagnosed (i.e., pre-existing before cancer diagnosis or post-acquired CVD after cancer diagnosis) as determined by self-report.”

Results:
Page 8:
• I stumble over the expression "a final analytical sample". It sounds like there are several. Is this the case?

‘Final analytic sample’ refers to the final sample retained after the sample selection process, as depicted in Figure 1, who were analyzed for the present study. We have clarified this by differentiating between the final analytic sample and the excluded sample in the Method section.

• Are there covariates with p>.20 included in the final model after variable selection? (line 6). Please clarify. Also, it is not clear from the results presented which covariates were included in the model.

Corrected. It should be P<.20. We have also revised the text to indicate the covariates retained through backward elimination in the Result section as well as in the note under the Table 3 and 4.

Page 9:
• Presenting the estimators of the complete model shown in Figure 2 in the appendix would give the reader the opportunity to look at the magnitudes of the estimators of the influencing factors and the confounders.

We have removed Figure 2 per request of earlier reviewers.

• Why are pre-existing CVD and post-acquired CVD independently associated with mortality? Please clarify "independently".

This has been rephrased to “In our study, we observed that both CVD status and PIR tertile were associated with all-cause mortality after multivariate adjustment.”

• In contrast to the presentation in Table 3, why is the lowest PIR mentioned as a reference in the text? This should be consistent.

This has been updated.

Table 3: Why are there two columns of hazard ratio? Are the HRs in the right-hand column based on models in a subpopulation, or were the PIRs combined for the middle and highest groups?

For Table 3, the first original column used the highest tertile poverty-income-ratio group as the reference versus the second used the middle tertile as the reference. For CVD status, the first column used no CVD as a reference, and the second used pre-existing CVD as the reference. We understand this can be confusing and take away from the message, so I have updated the table to only have 1 reference (highest-tertile) for PIR and no-CVD for CVD status.

Page 10:
• I cannot evaluate the results of the interaction analysis based on the data presented.

The first model (Table 3) examined the independent associations of PIR and CVD status with all-cause mortality without including an interaction term, as the presence of an interaction term would distort parameter estimates for the independent associations of PIR and CVD. Subsequently, the interaction term was added to the model and tested for significance, with the results reported in the Results section but not explicitly included in the table. “When assessing interaction terms between CVD and PIR, no statistically significant interactions were found (*P*>0.05).”

We have also clarified this in the note under Table 3 as “Hazard ratios are estimated from a Cox proportional hazard regression model, which includes both PIR and CVD status without an interaction term, while adjusting for age, sex, race/ethnicity, cancer type, marital status, smoking, alcohol consumption, physical activity, body mass index, and walking difficulty score that were retained by the backward elimination method (*P*<.20).”

• The statement that in every PIR tertile CVD increases the risk of mortality cannot be said with certainty with the presentation in Table 4. This is because the reference in the first model (column 5 of the table) is "No CVD in the highest PIR tertile" and applies to all combined PIR-CVD states. A stratified analysis might be more informative.

We revised the text to clarify the interpretation of the results from the model testing the combined associations of PIR and CVD status in Table 4. Additionally, we updated Table 4 by removing the results of additional risk comparison between the groups. Such additional comparisons of mortality risk using alternative reference categories is now presented in Supplement Table 2. For the analysis of the combined association, we estimated the hazard ratios of each group relative to all other groups. This approach enabled us to compare the risk of mortality by CVD status within each PIR, and vice versa, similar to stratified analyses. It also allowed us to make comparisons across different PIR-CVD groups. We have clarified this across the result and method sections as well as in the note under the Table 4.

Table 4. As with Table 3, it is unclear to me why different models were presented and on which study population they are based.

As with Table 3, we used alternative references with the same study population to emphasize the impact of PIR on all-cause mortality.

Discussion:
Where are the results for the statement that low income was independently associated with an increased risk of CVD? I have only seen models with all-cause mortality as the outcome variable. Table 2, on the other hand, does not show adjusted risk estimates but prevalences.

I have changed the wording throughout the manuscript. Due to the nature of the study population, we can only assess the prevalence of CVD. I have removed the word risk. In the future, this would be interesting to evaluate.

I also cannot understand the statement that low income is a stronger predictor of mortality than CVS when I look at Table 3 (page 13, lines 14-15).

Thank you for commenting on this. This is based on the similar HR between low-income (compared to high-income) and post-acquired CVD (compared to no CVD). The wording has been updated to make this clearer as this was also commented on by reviewer #1

Page 11:
Due to the lack of presentation of the influences of the covariates, the discussion cannot always be compared with the study results (e.g., with regard to the status of health insurance). The way it is presented in the discussion, and I can also imagine it, the lack of health insurance has an essential influence on general health. This aspect is discussed, but not substantiated with figures from the study in relation to the endpoint of all-cause mortality.

We have created and added supplemental tables to display the descriptive characteristics of the study population by CVD status. Additionally, we included the full Cox regression model, presenting estimated parameters for all study covariates retained in the model after the backward elimination method. These are Supplemental Table 1 and 2.

Please specify “higher income groups”.

The wording has been changed to refer to the highest PIR tertile (Page 10, Line 20-21)

I have noticed several typos and missing spaces. Here are a few:
Page 4, line 2: “.(15).”
Page 5, line 23: Shouldn’t it be “college or above” rather than “≥college or above”?
Page 10, line 13: “that that”
Page 11, line 5: “death(3).“
Page 18: Figure legends should be given for Figure 1 and Figure 2.

Thank you for commenting on this. We have gone through the paper and updated this. Figure legends are on Page 18 and the Figures are attached separately per request of the journal.

**Reviewer #3:**

Relevant and clearly structured manuscript. The topic is well introduced. However, some major and minor shortcomings should be addressed.
1. At the end of the introduction (p4, l. 2-12), a hypothesis is first formulated and then the questions and objectives of the study are stated. This passage of the manuscript is not clearly formulated and should be revised. The research question, hypothesis and aimes do not match. Specifically, the sentence on page 4, line 2 should be in more detail elaborated (e.g. reference to the limited information available, or reformulate the sentence in case no previous research has been done on the topic).

We have rewritten and reformatted this section. Additional references have been added.

“Cancer survivors exhibit a higher prevalence of cardiovascular risk factors, including obesity, hypertension, diabetes, and physical inactivity, compared to the general population [13, 14]. As a result, CVD becomes a significant cause of morbidity and mortality in cancer survivors. Several large studies support that most site-specific cancers have an increased risk of CVD compared to the general population [15]. Additionally, certain cancer therapies are linked to long-term cardiovascular effects such as coronary artery disease, heart failure, and vascular disease [16, 17]. However, less is known about the impact of income on cardiovascular risks and mortality rates among cancer survivors [18, 19].

To address this knowledge gap and evaluate how income disparities impact cardio-oncology outcomes, we performed an analysis using a nationally representative data, the National Health and Nutrition Examination Survey (NHANES), to provide insight into the relationship between income inequality, CVD, and all-cause mortality in cancer survivors. Our study aimed to 1) assess the prevalence of CVD and income equality among cancer survivors and investigate their associations, and 2) explore the role of income inequality as a factor associated between CVD status and all-cause mortality risk in cancer survivors.“

2. The study population is introduced as a repeated cross-sectional survey. In the section on the measurement of outcomes, it becomes clear that these observations contain retrospective information on CVD and are matched with mortality data in the future. This should be explained in the section on the study population and the follow-up time should be specified.

We agree with your comments. The linking of the NHANES data with mortality data makes this a clear retrospective cohort analysis for our secondary Aim. This is a cross-sectional analysis for Aim 1 and a retrospective cohort analysis for Aim 2.

I moved this section from our “Measurement of Outcomes” to the Study Population:

To evaluate all-cause mortality, our sample population was linked with public use linked mortality files associated with the NHANES. Mortality data included the mortality status of the NHANES participants through December 31, 2015, and follow-up time from the date of interview to the death or the end of the mortality period.

3. Figure 1 shows that more than 20% of the observations are not used due to missing observations. Are these missings systematic or random over the covariates?

A majority of excluded sample were due to missing in PIR variable. Thus, we tested the between-group differences in demographic characteristics and other key variables/study covariates available, and updated the results in the Method section to provide the relevant information about the excluded sample.

“The excluded sample (n = 642) was not significantly different from the final analytic sample in terms of age, gender, marital status, health insurance, BMI, cancer types, and CVD status. However, it did exhibit a significantly lower education level, a lower proportion of non-Hispanic White individuals, and a higher level of MVPA. “

4. Could you please explain the procedure you used to calculate a 12 year sample weight? (p.5, l.8,9) For which analysis did you use these sampling weights?

Revised clarifying the calculation of the 12-year sampling weight in ‘Statistical analysis’ section:

“A 12-year sampling weight was calculated based on published guidelines (25), by dividing the 2-year sampling weight from each cycle by the number of NHANES cycles combined to estimate the population level parameters in the US.”.

We applied sampling weights to all analyses and clarified this as follows:

“All analyses accounted for complex, multistage, and probability sampling scheme of the NHANES (e.g., oversampling of minority sub-groups, survey non-response, and post-stratification adjustment) using the SURVEY procedures in SAS v9.4 (SAS Institute, Cary, NC).”

5. The concept of PIR (Poverty-Income ratio) is introduced to measure income. Please refer to the literature on this concept and please justify why you use this measure for categorization. Specifically, some arguments regarding classification into 2 (e.g. in poverty or not in poverty) or more groups, and arguments regarding alternative measures (e.g. adjusted household income) would be desirable.

The poverty income ratio allows comparisons over time because it is continually revised to account for inflation, versus adjusted household income. This index was specifically developed by NHANES to account for inflation, costs of living in a geographic region, etc. and has been used across several publications. This has been updated in the methods section. Due to sample size and to maintain significance across groups, we divided these into tertiles.

6. Please describe your categories of the CVD measurement. What does 2+CVD mean?

An additional sentence was added to measurement of outcome “Individual breakdowns for CVD (coronary artery disease, heart failure, and stroke) were reported. Individuals with more than two types of CVD were reported as 2+ CVD.”

7. Please describe your weighting procedure in the statistical analysis section. Did you use weighting only for descriptive statistics or also for the regression analysis?

Yes, we calculated a 12-year sampling weight and used it for all data analyses to account for complex sampling design of the NHANES. All data analyses were done using SURVEY procedures in SAS for this purpose. We revised the manuscript to clarify this in the ‘Statistical Analysis’ section.

“All analyses accounted for complex, multistage, and probability sampling scheme of the NHANES (e.g., oversampling of minority sub-groups, survey non-response, and post-stratification adjustment) using the SURVEY procedures in SAS v9.4 (SAS Institute, Cary, NC). A 12-year sampling weight was calculated based on published guidelines (25), by dividing the 2-year sampling weight from each cycle by the number of NHANES cycles combined to estimate the population level parameters in the US.”

8. Please refer to the literature on the backward elimination procedure. Specifically, how did you choose the 0.2 cut-off value? Is there a literature supporting this choice? Do you mean on page 8, line 6: P-value<.20? (Caution: typo)

Yes. We meant a p-value <.20. Thank you for catching that.

Based on our understanding, there is no absolute threshold of P-value recommended for retaining study covariates when using the backward elimination method. The choice depends on various factors, including the total sample size, the number of study covariates, their potential role as influencing factors for either the independent variable (IV), dependent variable (DV), or both, and the parsimony of the model. Given the observational in nature of the NHANES, our goal was to develop a parsimonious prediction model while retaining potential covariates associated with all-cause mortality at a marginally significant level of 0.20 (Note: 'Marginally significant' is a subjective interpretation of the P-value applied only for model building in the present study). Although not perfect, this also could possibly alleviate potential selection bias for between-group comparisons (i.e., by PIR and/or CVD status). In the literature, there is still considerable inconsistency in selecting the threshold of P-value for backward elimination, ranging from 0.01 or lower to as high as 0.30 or higher.

In line with the reviewer’s suggestions, we have found several studies that subjectively define a P-value of 0.20 as a threshold for the backward elimination method.

<https://pubmed.ncbi.nlm.nih.gov/25430504/>

<https://pubmed.ncbi.nlm.nih.gov/27151893/>

<https://pubmed.ncbi.nlm.nih.gov/25415265/>

<https://pubmed.ncbi.nlm.nih.gov/15567629/>

And, although not ‘directly’ related to backward elimination for Cox regression model, in the study of Wang et al (2006) entitled “Determination of the Selection Statistics and Best Significance Level in Backward Stepwise Logistic Regression Qinggang Wang, John J. Koval,Catherine A. Mills & Kang-In David Lee”, the authors(s) recommend to use P-value between .20 and .40 as a stopping criterion for variable selection (https://www.tandfonline.com/doi/full/10.1080/03610910701723625).

9. Please explain your number of weighted cancer survivors (p8, l14). How can this number be interpreted?

Revised.

“The final analytic sample included 2,464 cancer survivors, which when weighted, represented 12,583,919 cancer survivors, equivalent to 7.91% of US adult’s ≥18 years in age.”

10. Please refer in the results section always to a table or figure (p8. l14-20)

A sentence has been added “Baseline demographics are reported in **Table 1**.”

11. Isn't backward elimination a way of reducing covariates in the model rather than part of the regression analysis? (p.9. l.15)

Correct. I have removed that part of the sentence to aid in clarity.

12. You use several cohorts (P.9, l.17)

This has been changed to study.

13. Please explain the findings of Table 3 before you refer to them.

This has been changed.

14. Tables 3 and 4 and Figure 2 are not self-explanatory. How can "weighted % of deaths" be interpreted? Are the HRs of PIR and CVD status (Table 3, Figure 2) estimated simultaneously in one model or in separate models? What is the meaning of the different models in Tables 3 and 4? Please explain the meaning of independent and combined in this context. Please explain which hypothesis you are testing in which table/model. Overall, the problems in (1) above continue here. This part of the manuscript is also unclear and should be revised.

Revised.

“weighted % of deaths” in the table 3 and 4 represents the population-level prevalence (%) of all-cause deaths among cancer survivors. This information has been added in the note under the tables. Table 3 presents the results of Cox regression analysis, including both PIR and CVD as primary predictors while adjusting for study covariates retained by backward elimination methods. We have clarified this in both the method and result sections. Additionally, the note under the table reiterates this information. We have also added more description of the models used to create Tables 3 and 4 in the method and result sections (see below). Table 4 is derived from a separate Cox regression model that includes a combined variable (PIR-by-CVD status consisting of nine sub-groups) as a primary predictor of all-cause mortality. To avoid any unnecessary confusion, we have removed additional Hazard Ratios (HRs) obtained with a alternative reference group, which are now presented in the supplement table. We have also clarified this in the note under the Table 4.

“A multivariate Cox proportional hazards regression model was constructed predicting the risk of all-cause mortality based on PIR and CVD status among cancer survivors. We first fitted a model including both PIR and CVD status as primary independent variables predicting the risk of all-cause mortality. A linear trend of the risk across PIR tertiles was tested using orthogonal polynomial contrasting, and the interaction effects between PIR and CVD status were also examined. The risk of all-cause mortality was presented using hazard ratios (HR) relative to the reference category (lowest tertile for PIR; and no-CVD for CVD status). The second model included the combined variable of PIR and CVD consisting of 9 combined groups (three PIR tertiles by three CVD status) to test their joint association with mortality risk. The risk of all-cause mortality was estimated relative to the highest PIR tertile/no-CVD group as the primary reference. Additionally, HRs relative to different groups were reported as a supplement.”

15. The text and the numbers related to Table 3 do not match (page 9, lines 19-22). Please, correct them.

This has been changed.

In sum I come to the conclusion, that the paper is unsuitable for publication in its present form. However, the study itself shows sufficient potential for publication. Therefore, the authors are encouraged to resubmit a revised version.

Thank you for giving is the opportunity to revise this paper.
