# Supplementary material for: Association of poverty-income ratio with cardiovascular disease and mortality in cancer survivors in the United States
Source: PLoS One. 2024 Jul 5;19(7):e0300154. doi: 10.1371/journal.pone.0300154 (PMC11226125; doi:10.1371/journal.pone.0300154)
Supplement: Attachment — Submitted filename: 1_2. Response letter_2nd revision.docx [file pone.0300154.s003.docx]

**Dear Reviewers,**

**Thank you so much for taking the time to give us feedback. We appreciate the opportunity. Our revisions are addressed below:**

**----**

Reviewer Comments:

Comment: Abstract: I think it is necessary to define the study participants and the related used data base in more detail. This will affect the judgement of the readers about the possible bias and confounders.

Thank you for your comment. We have expanded the abstract methods section to include this:

This study utilized nationally representative data from the National Health and Nutrition Examination Survey (NHANES), a cross-sectional survey evaluating the health and nutritional status of the US population. Our study included NHANES participants aged ≥20 years from 2003-2014, who self-reported a history of cancer. We evaluated the association between income level, prevalence of CVD, and all-cause mortality. All-cause mortality data was obtained through public use mortality files. Income level was assessed by poverty-income ratio (PIR) that was calculated by dividing family (or individual) income by poverty guideline. We used multivariable-adjusted Cox proportional hazard models through a backward elimination method to evaluate associations between PIR, CVD, and all-cause mortality in cancer survivors.

Comment: Material & method: there are a number of issues to be noticed in this section:

Thank you. We have addressed each of the questions below:

Comment: I think it is essential to show the reliability of the question used for the diagnosis of cancer in recruited patients. Another issue is the matter of the time of cancer diagnosis which shows the time to the event (outcome) of the study.

Page 11, Line 2-5. I have updated this section to address the comment.

Self-reported behavior and medical history, including history of cancer, may entail misclassification and/or underreporting. Although NHANES is regarded as a valid assessment tool [11, 52], with several recent publications using self-reported cancer history [53,54]. For this study, the time of cancer diagnosis was not the primary outcome; instead, it was utilized as one of the study covariates adjusted in the model(s). The main outcome of the present study was mortality. Nevertheless, potential self-reported bias regarding the time of cancer diagnosis was acknowledged in the discussion section.

Comment: Please define the inclusion and exclusion criteria in more detail. It seems more criteria have been used for recruiting the participants.

Page 4, Line 8-12 is now updated. “Our analysis included all adult participants in NHANES with publicly available data and a history of cancer. For this study, history of cancer (aged ≥18 years) was identified based on self-reported medical history data.”

Comment: Please show how the parameters used for the calculation of PIR have been measured.

This section has also been updated, starting at Page 5, line 10:

During a household interview, NHANES participants were asked to report total family income in the previous calendar year. This total family income is then divided by the Department of Health and Human Services (HHS) poverty guidelines threshold, specific to family size, calendar year, and state. The PIR index, developed by NHANES, accounts for regional cost of living and annual inflation. PIR has been extensively validated as a measure of income and has been documented in various studies [11, 25].

The PIR values ranged from 0 to 5 (≥ 5 due to disclosure concerns). A PIR value of 0 corresponds to no income, 1 indicates a family income at 100% of the federal poverty level, and 5 corresponds to ≥5 times the federal poverty level.

Comment: The matter of measuring method of the covariates remains an important issue in the measurement of covariates. Another issue to be noticed is the probable collinearity of these variables with exposure of interest (PIR), which may cause problems for the analysis of the data.

Response: Thank you so much for your insightful comments. In our study, the covariates were measured using standardized questionnaires and/or protocols set by the National Health and Nutrition Examination Surveys. These are well-validated, including the PIR measures. We acknowledge the potential for collinearity between the covariates and PIR, which is a common challenge with these studies. We used multivariate-adjusted Cox models with backward elimination to retain only covariates that contribute independently to the model, which may reduce the impact of collinearity. We further tested for potential bias due to multicollinearity in the final analytic model(s) based on variance inflation factor (VIF) and found no evidence of multicollinearity (VIF < 5).

Comment: Showing the reliability and validity of measuring the outcomes of interest, is essential. Results and discussion: according to the above-mentioned comments these sections are not justifiable.

Thank you again for taking the time to allow us to revise this manuscript and for the feedback. One of the major limitations with NHANES and these study times, which is mentioned in the limitations section, is the self-reported nature of the variables and the all-cause mortality data. See limitations section:

“Self-reported behavior and medical history, including history of cancer, may entail misclassification and/or underreporting. Although NHANES is regarded as a valid assessment tool [11, 52], with several recent publications using self-reported cancer history [53, 54].” …. “All-cause mortality in cancer survivors encompasses both cancer-related mortality and non-cancer related mortality. Income may differentially impact different cancer subtypes and mortality risks, with most studies reporting that low household income is associated with worse outcomes [53, 54]. Unfortunately, due to the limited sample size, we were unable to broaden our scope to include cause-specific mortality, including CVD-specific mortality. However, several large studies have reported that CVD is the most common cause of non-cancer death, likely secondary to cardiotoxicity [55, 56], allowing us to highlight that income level is an important risk measure for adverse health outcomes in this population.”

Additionally, these same outcomes have been used in several recently published studies:

- Fishman EI, Steeves JA, Zipunnikov V, Koster A, Berrigan D, Harris TA, Murphy R. Association between Objectively Measured Physical Activity and Mortality in NHANES. Med Sci Sports Exerc. 2016 Jul;48(7):1303-11. doi: 10.1249/MSS.0000000000000885. PMID: 26848889; PMCID: PMC4911242.
- Schmid D, Ricci C, Leitzmann MF. Associations of objectively assessed physical activity and sedentary time with all-cause mortality in US adults: the NHANES study. PLoS One. 2015 Mar 13;10(3):e0119591. doi: 10.1371/journal.pone.0119591. PMID: 25768112; PMCID: PMC4358950.
- Liang KW, Lee CL, Liu WJ. Lower All-Cause Mortality for Coronary Heart or Stroke Patients Who Adhere Better to Mediterranean Diet-An NHANES Analysis. Nutrients. 2022 Aug 5;14(15):3203. doi: 10.3390/nu14153203. PMID: 35956379; PMCID: PMC9370677.
- Moonesinghe R, Yang Q, Zhang Z, Khoury MJ. Prevalence and Cardiovascular Health Impact of Family History of Premature Heart Disease in the United States: Analysis of the National Health and Nutrition Examination Survey, 2007-2014. J Am Heart Assoc. 2019 Jul 16;8(14):e012364. doi: 10.1161/JAHA.119.012364. Epub 2019 Jul 13. PMID: 31303097; PMCID: PMC6662130.

Thank you again.
