# Supplementary material for: Association of poverty-income ratio with cardiovascular disease and mortality in cancer survivors in the United States
Source: PLoS One. 2024 Jul 5;19(7):e0300154. doi: 10.1371/journal.pone.0300154 (PMC11226125; doi:10.1371/journal.pone.0300154)
Supplement: Attachment — Submitted filename: 1_2. Response letter_3rd revision.docx [file pone.0300154.s004.docx]

**Dear Plos One Reviewers,**

**Thank you again taking the time to give us feedback. We have made revisions below to address your comments.**

**Sincerely,**

**Youngdeok Kim, PhD**

**----**

Reviewer Comments:

Comment: **It is very important to show the reliability of measuring a number of variables including measurement of economic status of the recruited participants which are based on their response.**

Response: Thank you for pointing this out. Under study population on Page 4, we write “The NHANES is a cross-sectional survey evaluating the health and nutritional status of the US population in 2-year cycles based on nationally representative samples selected by a complex sampling design [20]. The NHANES examines various health-related outcomes through household interviews, physical examinations, and laboratory tests. The survey protocol of the NHANES was approved by the Research Ethics Review Board at the NCHS. Our analysis included all adult participants in NHANES with publicly available data and a history of cancer. For this study, history of cancer (aged ≥18 years) was identified based on self-reported medical history data. We considered cancer survivors to be those individuals who had been previously diagnosed with cancer and answered “yes” to the question, “Has a doctor ever told you that you had cancer or malignancy?” Cancer sub-types were assessed through response to the question “What kind of cancer?”, and respondents were able to enter up to 3 kinds. Additionally, data on age at cancer diagnosis was collected through a separate survey question [21, 22]. “ On page 5, under “Income measures”, we have a sentence “PIR has been extensively validated as a measure of income and has been documented in various studies [11, 25].” Similar findings are written under the outcome values. These cross-sectional values have been validated across a number of studies, including in PlosOne, i.e., PMC6957172 and PMC2737140.

**Comment: According to what is mentioned in the material and method section, the study is a historical cohort one, in that case expression of the results shown in the first paragraph of the result section is meaningless. The two study groups are those with higher and lower PIR tertile not the CVD ones.**

Response: Thank you for your insightful comment. I have added to the statistical methods to clarify that our groups of interest are the PIR tertiles, as you mentioned. Page 5, Statistical Analysis “Descriptive characteristics of study variables among cancer survivors were calculated using mean or percentage along with 95% confidence intervals for continuous and categorical variables, respectively by PIR tertile.” Additionally, the results section has been changed to reflect an emphasis on PIR tertile. “There were 821 (weighted 1.78%) participants in the lowest PIR tertile, 815 (weighted 2.48%) in the middle tertile, and 828 (weighted 3.65%) in the highest PIR tertile”

**Comment: Because of the nature of the study, it is necessary to show how probable selection biases such as immortal time bias, have been managed in this study. In fact, the differences shown in two groups of PIR status might have been related to the general condition differences of these two groups not to the proposed variable of PIR. This is shown somehow in table 1. In this case the conclusions may need to be revised totally.**

Response: Thank you for your insightful comment. Unfortunately, NHANES is a cross-sectional study linked solely to follow-up mortality data, which means it doesn't repeatedly measure exposure status over time. This limitation restricts our ability to estimate time-dependent effects of exposure variables. We have acknowledged this inherent constraint in the survey data within the discussion sections.

Additionally, all study variables, including sample characteristics presented in Table 1, were considered as potential covariates during the development of our final analytic models. Multiple variables associated with PIR were subsequently retained and adjusted in the statistical model(s). We have clarified this process in the method section and updated the results/tables/discussion in the previous revision.

Lastly, our primary aim was to examine the prevalence of CVDs among cancer survivors, given the cross-sectional nature of NHANES. It is true that immortal time bias and other survival-related biases could influence the results of our secondary aim, and we have acknowledged them in the discussion section.

**Comment: Another issue to be noticed in this study is the fact that measurement of the PIR has been conducted after the diagnosis of both cancer and CVD, which may not be a correct reflection of the PIR status of the start of the study.**

Response: Thank you for the helpful comment. We would like to clarify that, for the survival analysis predicting the risk of mortality, the starting point of the study was not the diagnosis of cancer or CVD but the time when individuals participated in the NHANES survey. We have also made this clarification in the method section:

“Mortality data included the mortality status of the NHANES participants through December 31st, 2015, and follow-up time from the date of interview to the death or the end of the mortality period.”

Additionally, minor wording changes have been made throughout the manuscript.

Thank you so much once again for the opportunity.
